# Supplementary material for: Nuclear lamina strain states revealed by intermolecular force biosensor
Source: Nat Commun. 2023 Jun 30;14:3867. doi: 10.1038/s41467-023-39563-6 (PMC10313699; doi:10.1038/s41467-023-39563-6)
Supplement: Supplementary file 1 — Supplementary Information [file 41467_2023_39563_MOESM1_ESM.pdf]

Nuclear lamina strain states revealed by intermolecular force biosensor

Brooke E. Danielsson[1], Bobin George Abraham[2], Elina Mäntylä[2], Jolene I. Cabe[1], Carl R Mayer[1], Anna Rekonen[2], Frans Ek[2], Daniel E. Conway[3,4], Teemu O. Ihalainen[2,5]

1) Department of Biomedical Engineering, Virginia Commonwealth University, Richmond, Virginia, USA
2) BioMediTech, Faculty of Medicine and Health Technology, Tampere University, Tampere, Finland
3) Department of Biomedical Engineering, The Ohio State University, Columbus, Ohio USA
4) The Ohio State University and Arthur G. James Comprehensive Cancer Center, The Ohio State University, Columbus, Ohio USA
5) Tampere Institute for Advanced Study, Tampere University, Tampere, Finland

Supplementary material:

Supplementary figures and figure legends:

Supplementary Figure S1. Lamin-SS structure and its effect on lamin A/C expression

Supplementary Figure S2. Characterization of the lamin nanobody epitope region in lamin A/C

Supplementary Figure S3. Lamin filament assembly and Lamin-SS binding to lamin network

Supplementary Figure S4. Characterization of LMNA KO cells and Lamin-SS distribution and FRET efficiency in the LMNA KO cells

Supplementary Figure S5. Fluorescence recovery after photobleaching (FRAP) experiments of Lamin-SS and Lamin-TM binding to nuclear lamina

Supplementary Figure S6. Schematic representation of the simulated FRAP experiment and reactions

Supplementary Figure S7. Effect of actin cytoskeleton disruption on Lamin-SS FRET

Supplementary Figure S8. Effect of ROCK-pathway inhibition on nucleus morphology

Supplementary Figure S9. Effect of histone deacetylase inhibition on nucleus morphology

Supplementary Figure S10. Nuclear lamina organization after chromatin relaxation

Supplementary Figure S11. Fluorescence recovery after photobleaching (FRAP) experiments of Lamin-SS after TSA treatment

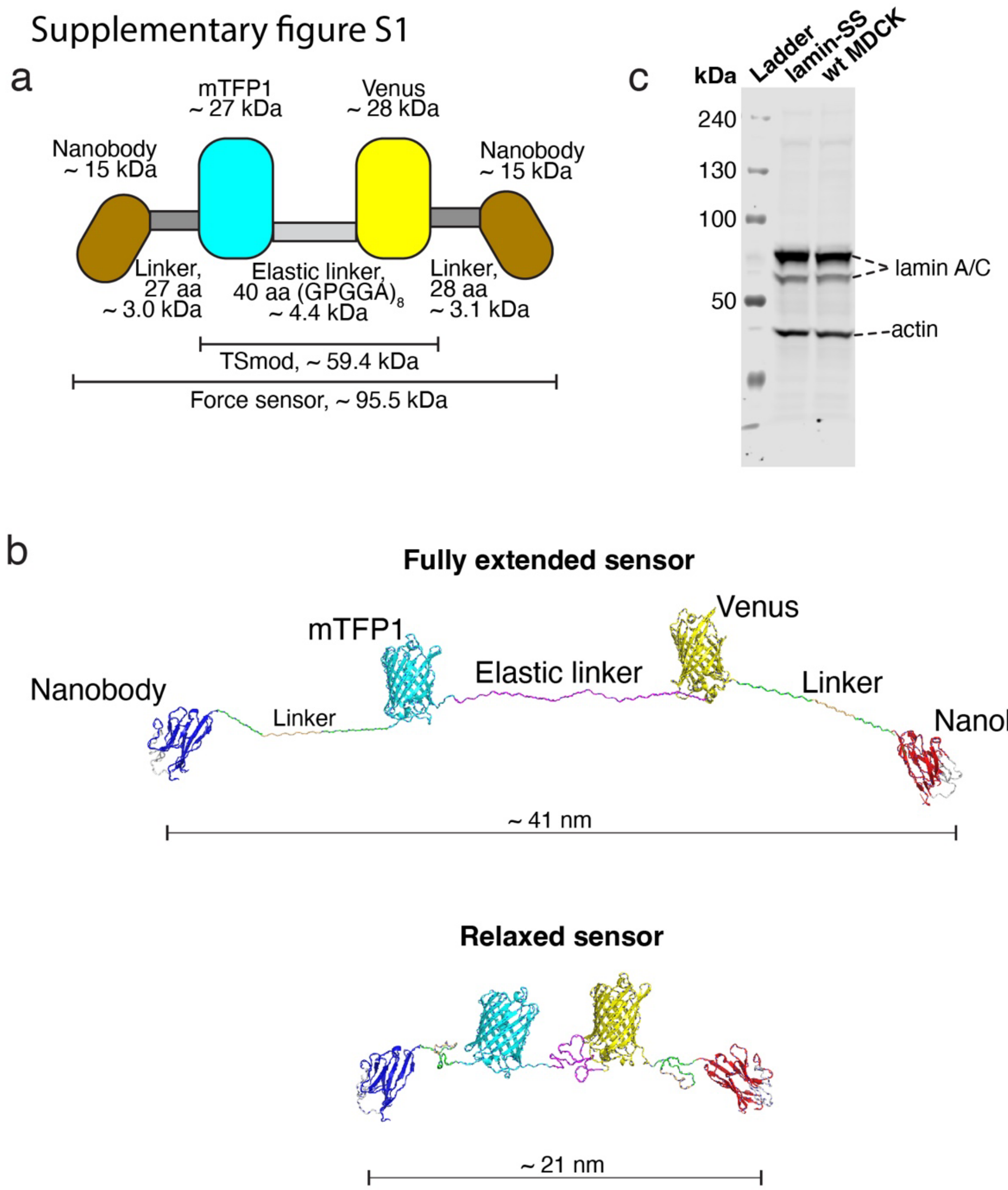

Supplementary Figure S1. Lamin-SS structure and its effect on lamin A/C expression

a, Schematic representation of the Lamin-SS. TSmod force sensing module is flanked by linkers and lamin nanobodies. **b,** Simulated Lamin-SS size in extended and compacted i.e., relaxed forms. **c,** Representative WB of lamin A/C expression in Lamin-SS expressing and wild type (WT) MDCK cells (n=2 biological replicates).

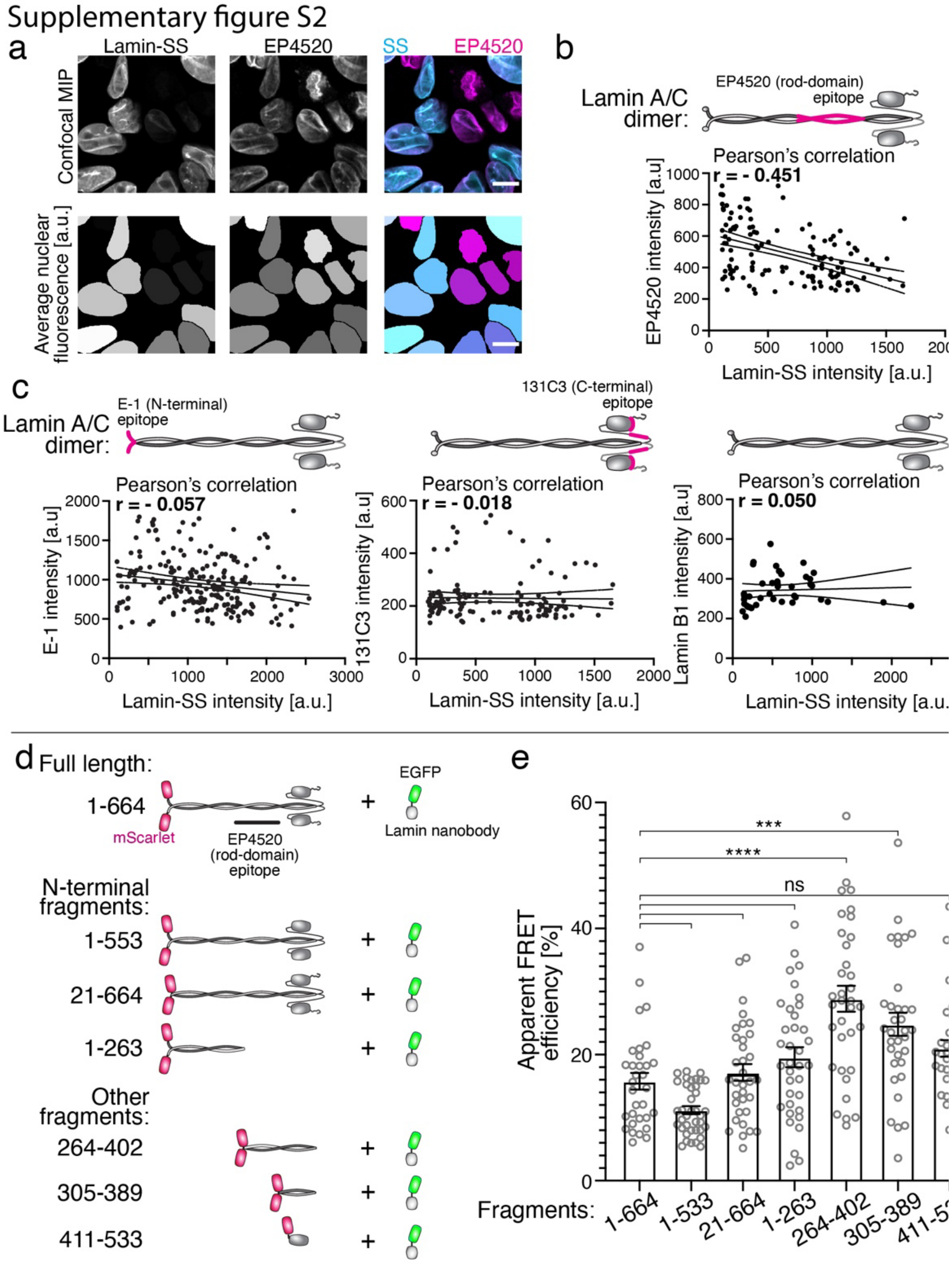

Supplementary Figure S2. Characterization of the lamin nanobody epitope region in lamin A/C

a, Representative confocal maximum intensity projections showing Lamin-SS signal together with immunolabeled lamin A/C (EP4520, lamin A/C rod-domain antibody) (top) and the average nuclear fluorescence of the dyes (bottom). Scale bars, 10 µm. **b,** Scatter-plot of nuclear lamin-SS and EP4520 antibody intensities showing lines and errors presented as dotted lines depicting the best-fit trendline and the 95 % confidence interval of the linear regression, respectively, and their corresponding Pearson's correlation coefficient (n=130 cells, 3 biological replicates). **c,** Scatter-

plots showing nuclear intensities of two lamin A/C (E-1 and 131C3) or lamin B1 antibodies showing lines and errors presented as dotted lines depicting the best-fit trendline and the 95 % confidence interval of the linear regression, respectively, and their corresponding Pearson's correlations. Pink color on the schematic representations of the lamin A/C dimer indicates the respective binding sites of the antibodies. (n=208, 130 and 41 cells, respectively, 3 biological replicates). **d,** Schematic representation of the used lamin A/C truncates and the localization of the EP4520 epitope in the construct. **e,** Measured FRET (acceptor photobleaching method) (mean ± SEM) between the EGFP-tagged lamin nanobody and the expressed lamin A/C truncates in LMNA KO cells (n=242 cells (32-38 per construct), 2 biological replicates). Ordinary one-way ANOVA, multiple comparisons, (****) $p<0.0001$, (***) $p=0.0006$, df=6.

Supplementary figure S3

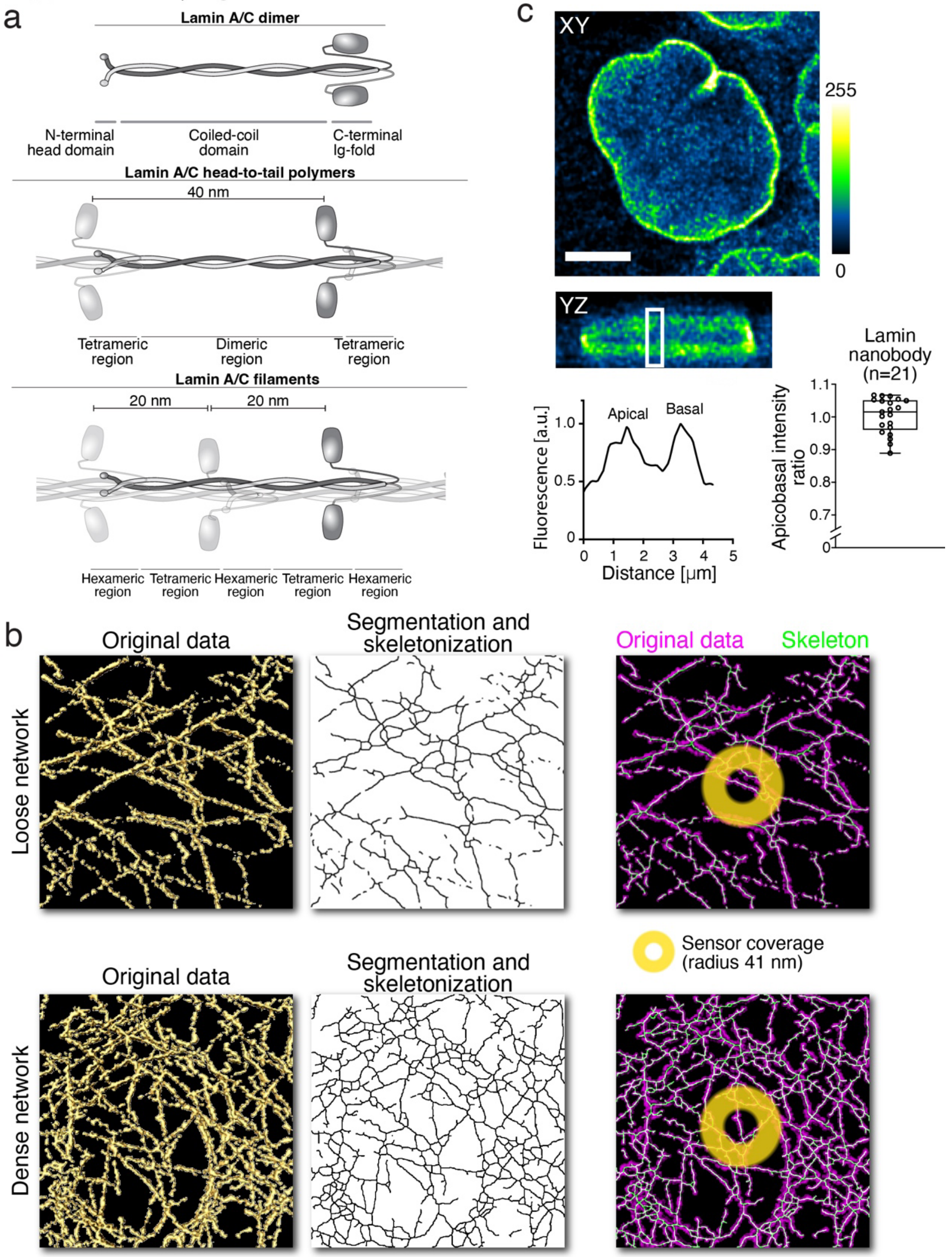

Supplementary Figure S3. Lamin filament assembly, Lamin-SS binding to the nuclear lamina, and modelled Lamin-SS binding to lamin network

a, Lamin filament assembly and corresponding periodicity of the filament. **b,** Original electron microscopy data of the lamina network (Turgay et al. *Nature* 543, 261–264 (2017)), image size

330 nm x 330 nm (left), segmented and skeletonized data (middle), and binding radius of the Lamin-SS in the nuclear lamina (right). The yellow area corresponds to the radius of the sensor (21 – 41 nm, see supplementary figure S1). **c,** Representatitive single confocal XY- and YZ-sections of Lamin-SS expressing cell (top). Scale bar 5 µm. Apicobasal fluorescence profile (white box) of lamin-SS indicates even distribution to basal and apical sides of the nuclei (lower left). Quantified apicobasal intensity ratios of the Lamin-SS (box from 25th to 75th percentile, median, whiskers from min to max, n=21 nuclei, 2 biological replicates) (lower right).

Supplementary figure S4

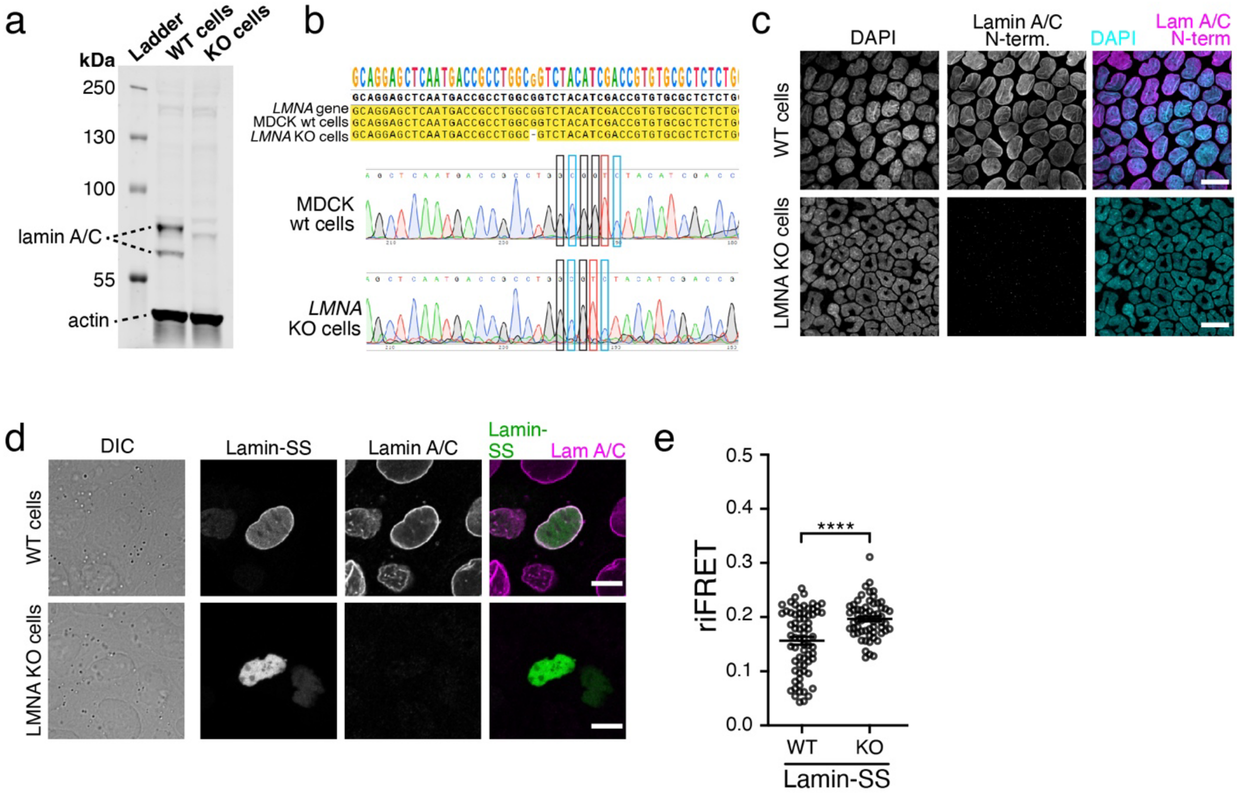

Supplementary Figure S4. Characterization of LMNA KO cells and Lamin-SS distribution and FRET efficiency in the LMNA KO cells

a, Immunoblot analysis of lamin A/C in MDCK Wild-type (WT)and LMNA KO cells showing successful knockout of the LMNA gene in KO cells (2 biological replicates). **b,** Sequencing of the LMNA gene in LMNA KO cells showing a frameshift mutation in the LMNA gene. **c,** Immunolabeling of LMNA KO and WT cells with a N-terminally binding lamin A/C antibody (E-1) showing undetectable amounts of lamin A/C in the KO cells (n=2 biological replicates). Scale bars 20 µm. **d,** WT and LMNA KO cells transiently transfected with Lamin-SS. In LMNA KO cells Lamin-SS localization to nuclear lamina is lost. Scale bars, 10 µm. **e,** Quantified riFRET efficiency (mean ± standard error of the mean) of Lamin-SS in WT and LMNA KO cells (n=69 and n=59 cells for WT and KO cell lines, respectively, 2 biological replicates). Unpaired two-tailed Student´s T-test ((****) $p \leq 0.0001$, t=4.64, df=126).

Supplementary figure S5

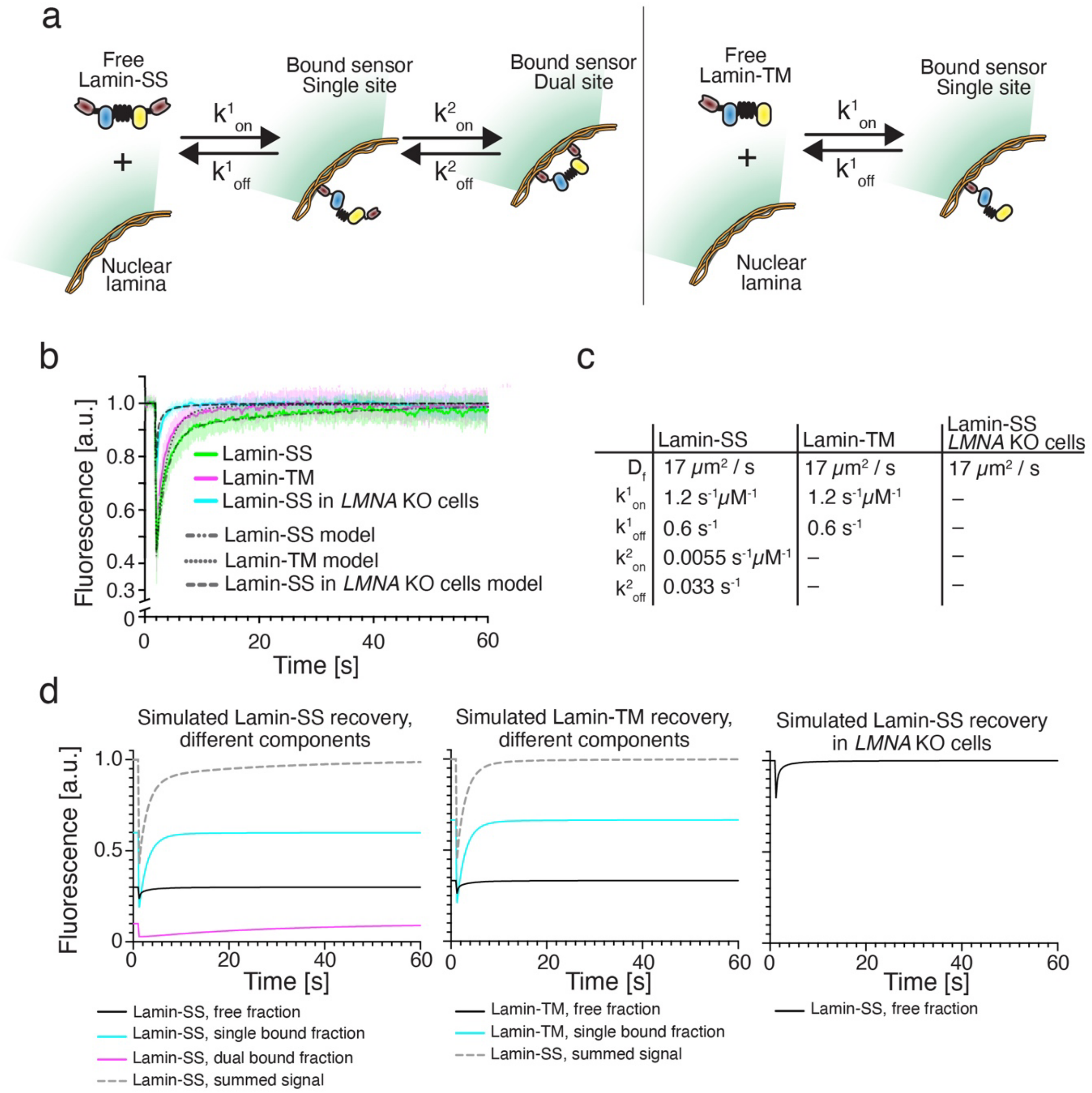

	Lamin-SS	Lamin-TM	Lamin-SS *LMNA* KO cells
D_f	17 μm^2 / s	17 μm^2 / s	17 μm^2 / s
k^1_{on}	1.2 $s^{-1}\mu M^{-1}$	1.2 $s^{-1}\mu M^{-1}$	–
k^1_{off}	0.6 s^{-1}	0.6 s^{-1}	–
k^2_{on}	0.0055 $s^{-1}\mu M^{-1}$	–	–
k^2_{off}	0.033 s^{-1}	–	–

Supplementary Figure S5. Fluorescence recovery after photobleaching (FRAP)experiments of Lamin-SS and Lamin-TM binding to nuclear lamina

a, Model of the Lamin-SS and Lamin-TM binding to nuclear lamina. The binding of Lamin-SS is assumed to proceed sequentially, from the binding of the first nanobody (single site) to the binding of the other (dual site). The release of the sensor from the lamina is modeled to proceed in reverse. Lamin-TM binding is limited to the single-site binding. **b,** Simulated recovery data together with the measured recoveries, see also main Figure 1. **c,** Free diffusion coefficients, and binding pseudo on-rate and off-rate of Lamin-SS and Lamin-TM used in the simulations shown in b. **d,** Individual components of the simulated recoveries for Lamin-SS (in WT and KO cells) and Lamin-TM (in WT cells).

Supplementary figure S6

a

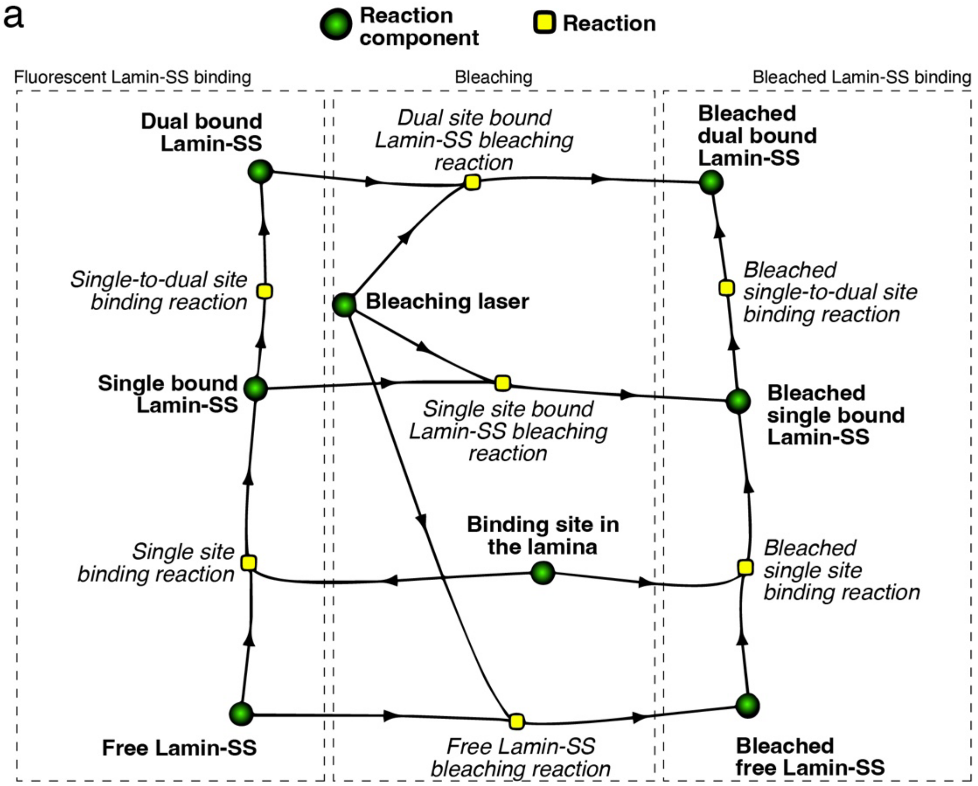

b

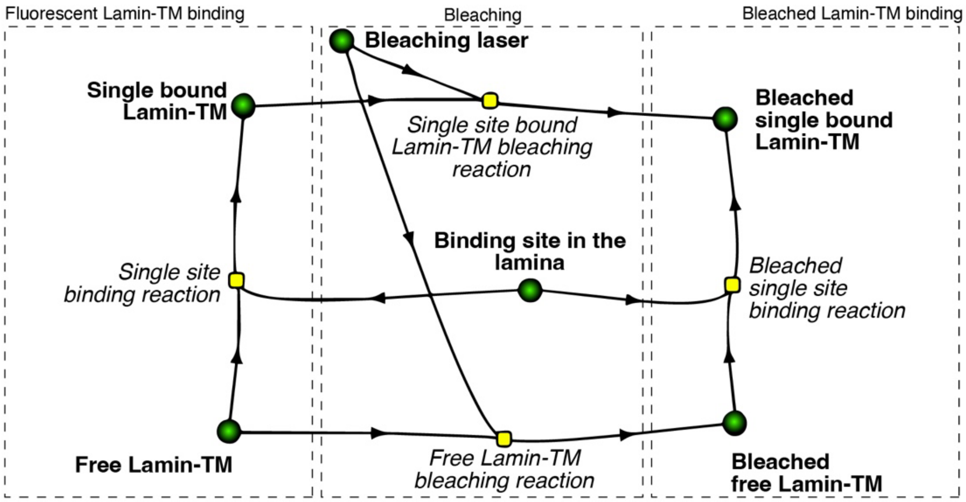

Supplementary Figure S6. Schematic representation of the simulated FRAP experiment and reactions

a, Reactions of Lamins-SS during FRAP experiment. Freely diffusing Lamin-SS interacts with a binding site in the nuclear lamina (single site binding reaction) yielding ingle-bound Lamin-SS. This can further lead to a dual-bound Lamin-SS (single-to-dual site binding reaction) or release of the sensor and binding site (reverse of single site binding reaction). Dual-bound Lamin-SS can be released by reverse reaction leading into single-bound Lamin-SS (reverse of single-to-dual site binding reaction). Bleaching is simulated by a local reaction between the bleaching laser, free Lamin-SS, single-bound Lamin-SS, and dual-bound Lamin-SS. The bleaching leads to the appearance of bleached species of Lamin-SS. **b,** Reactions of Lamins-TM during FRAP experiment. Truncated sensor binding is limited to the reaction between free Lamin-TM and the binding site in the lamina, yielding single-bound Lamin-TM (single-site binding reaction). Similarly, as Lamin-SS, truncated Lamin-TM sensor release leads to free Lamin-TM and binding site in the lamina (reverse of single-site binding reaction). Fluorescent Lamin-TM molecules are bleached in the bleaching reactions with the bleaching laser, yielding bleached single-bound Lamin-TM and bleached free Lamin-TM.

Supplemental figure S7

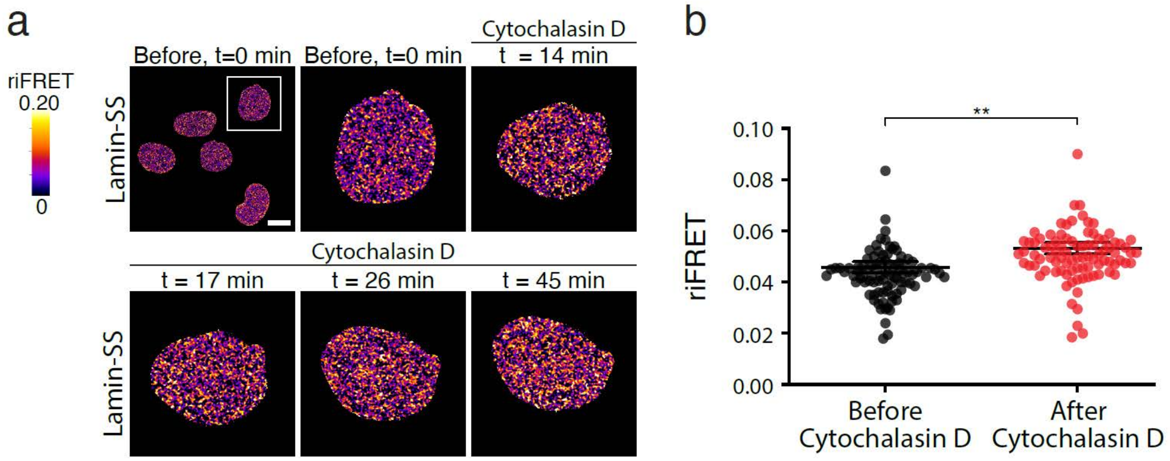

Supplementary Figure S7. The effect of actin cytoskeleton disruption on Lamin-SS FRET

a, Live cell imaging of Lamin-SS riFRET ratio during actin cytoskeleton disruption with cytochalasin D (10 µg/mL, added at 3 min timepoint). Scale bar 5 µm. Blow-up images of single nucleus indicate an increase in the riFRET. **b,** Quantified change in the riFRET (mean ± SEM) after 45 min of cytochalasin treatment (n=92 cells, 2 biological replicates). Paired two-tailed Student´s t-test ((*) p=0.019, t=2.36, df=182).

Supplementary figure S8

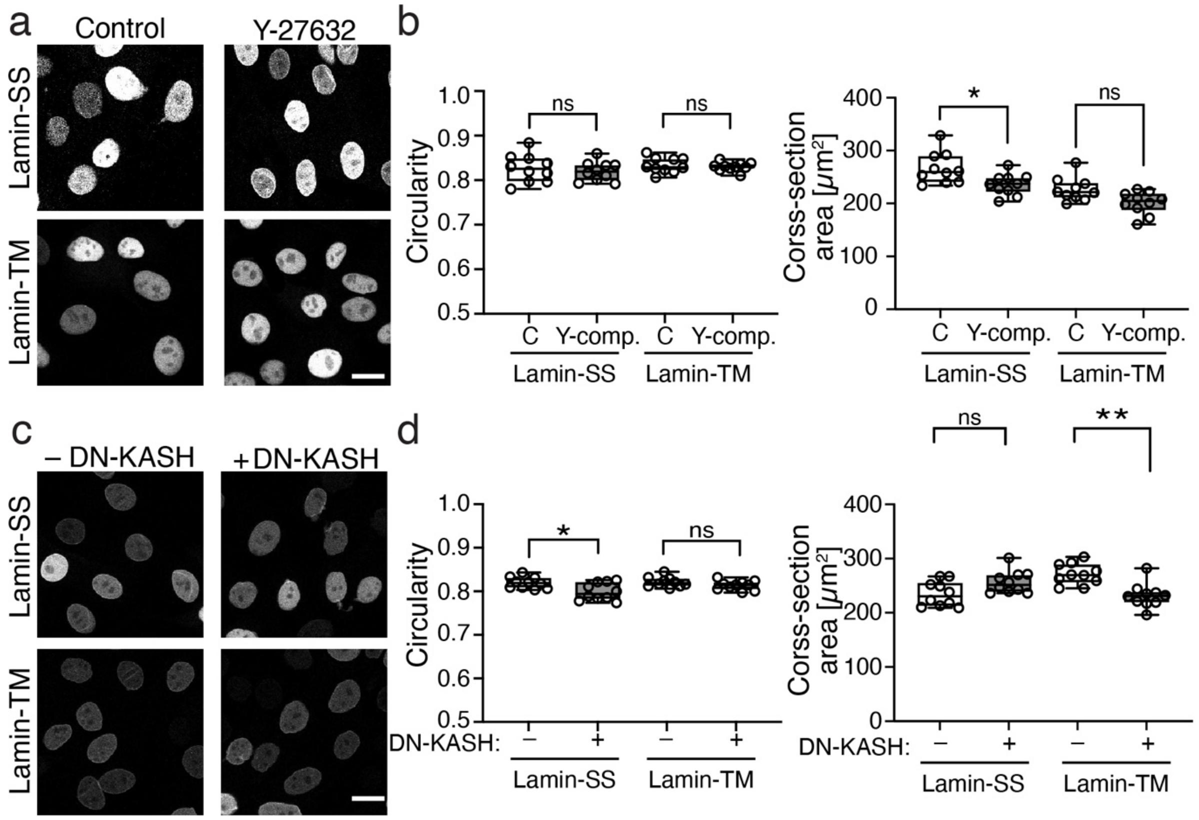

Supplementary Figure S8. The effect of ROCK-pathway inhibition and LINC-complex disruption on nucleus morphology

a, Representative single confocal sections of Lamin-SS and Lamin-TM expressing cells in control conditions or under ROCK inhibition (Y-27632, 50 µM, 1 h). Scale bar 20 µm. **b,** Quantification of nuclear circularity and nucleus cross-sectional area in the untreated control (C) and ROCK-pathway inhibited (Y-27632) Lamin-SS and Lamin-TM -expressing MDCK cells (box from 25th to 75th percentile, median, whiskers from min to max, n=5 fields, 2 biological replicates). Ordinary one-way ANOVA, multiple comparisons (circularity p = 0.904 (SS), p=0.982 (TM), cross-sectional area p=0.034 (SS), p=0.090 (TM), df=36). **c,** Representative single confocal sections of Lamin-SS and Lamin-TM expressing cells in control conditions or when LINC complexes are disrupted (DN-KASH expression, 24 h). Scale bar 20 µm. **d,** Quantification of nuclear circularity and nucleus cross-sectional area (mean ± SEM) in the untreated control (C) and LINC-complex disrupted Lamin-SS and Lamin-TM -expressing MDCK cells (box from 25th to 75th percentile, median, whiskers from min to max, n=10 fields, 2 biological replicates). Ordinary one-way ANOVA, multiple comparisons (circularity p = 0.0166 (SS), p = 0.6079 (TM), cross-sectional area p=0.1248 (SS), p=0.0010 (TM), df=36).

Supplementary figure S9

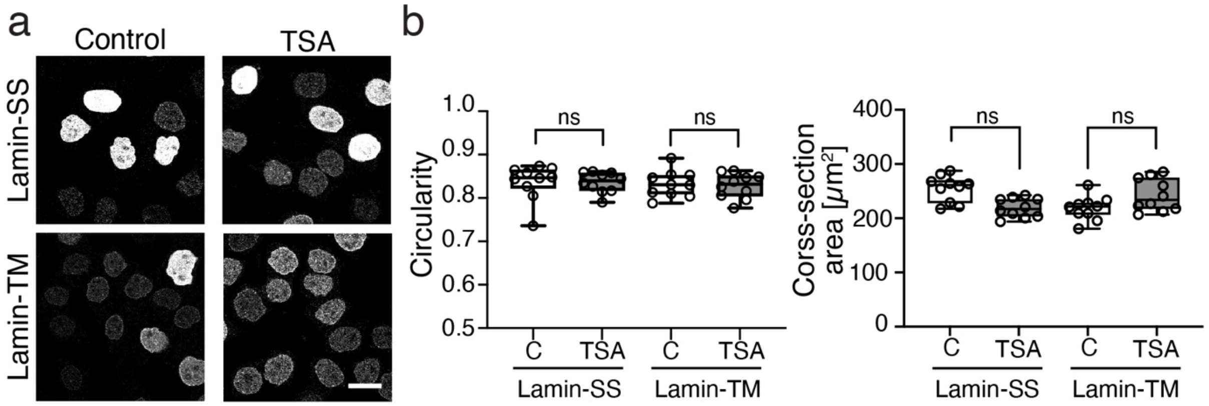

Supplementary Figure S9. The effect of histone deacetylase inhibition on nucleus morphology a, Representative single confocal sections of Lamin-SS and Lamin-TM expressing cells in control conditions (C) or under histone deacetylase inhibition (trichostatin A, TSA, 200 nM, 4 h). Scale bar 20 µm. **b,** Quantification of nuclear circularity and nucleus cross-sectional area (mean ± SEM) in the nontreated control (C) and the TSA-treated Lamin-SS and Lamin-TM -expressing cells (box from 25th to 75th percentile, median, whiskers from min to max, n= 10 fields, 2 biological replicates). Ordinary one-way ANOVA, multiple comparsions (circularity $p > 0.999$ (SS), $p=0.999$ (TM), cross-sectional area $p=0.353$ (SS), $p=0.743$ (TM), $df=54$)

Supplementary figure S10

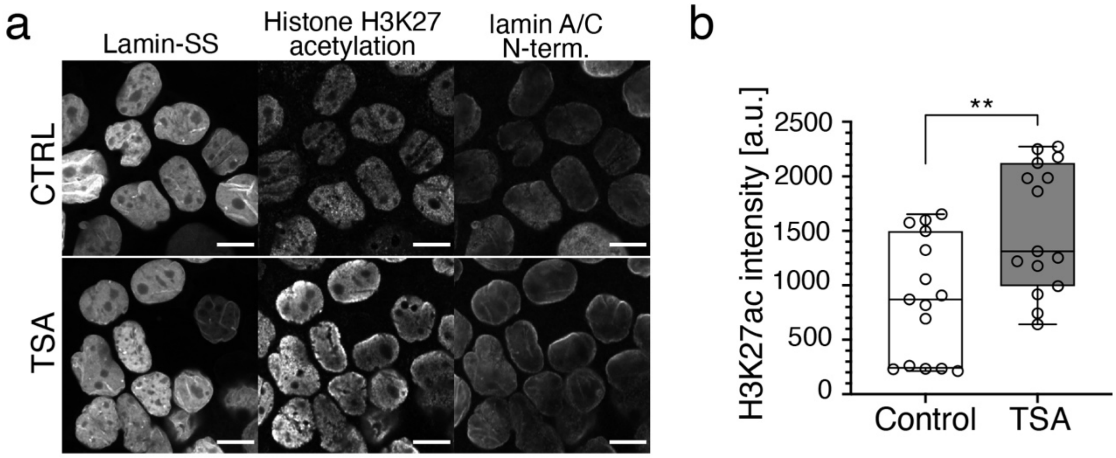

Supplementary Figure S10. Nuclear lamina organization after chromatin relaxation

a, Confocal maximum intensity z-projection images of nontreated control (upper panels) and trichostatin A (TSA) -treated (600 nM, 4 h, lower panels) Lamin-SS expressing cells immunolabeled against histone H3 lysine 27 acetylation (H3K27) and N-terminal part of lamin A/C (E-1). Scale bars, 10 µm. **b,** Quantification of nuclear fluorescence intensity of H3K27 acetylation labeling in nontreated control and TSA-treated cells (box from 25th to 75th percentile, median, whiskers form min to max n=15 fields, respectively, 3 biological replicates). Unpaired two-tailed Student´s t-test (** p=0.004, t=3.56, df=14).

Supplementary figure S11

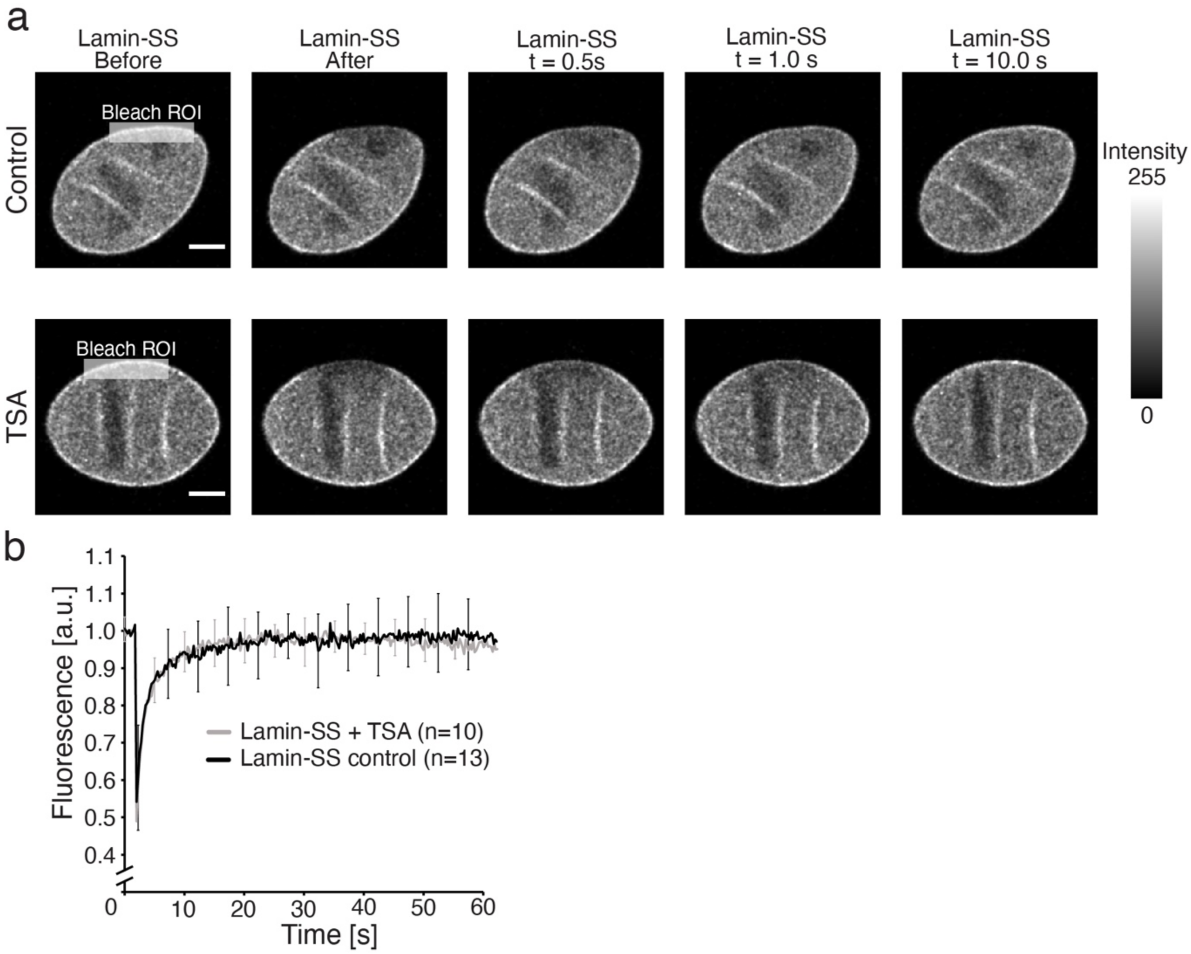

Supplementary Figure S11. Fluorescence recovery after photobleaching (FRAP) experiments of Lamin-SS after TSA treatment

a, FRAP experiment with Lamin-SS expressing cells in nontreated control cells (upper panel) and after their trichostatin A (600 nM, 4h). The bleached region of interest (ROI) is marked in the image. Scale bar 5 µm. **b,** Quantified and normalized fluorescence recoveries (mean ± standard deviation) of Lamin-SS in control and TSA-treated cells (n=10 and 13 cells, respectively, 2 biological replicates).
