# Supplementary material for: Nuclear lamina strain states revealed by intermolecular force biosensor
Source: Nat Commun. 2023 Jun 30;14:3867. doi: 10.1038/s41467-023-39563-6 (PMC10313699; doi:10.1038/s41467-023-39563-6)
Supplement: Supplementary file 3 — Description of Additional Supplementary Files [file 41467_2023_39563_MOESM3_ESM.pdf]

Description of Additional Supplementary Files

Supplementary Data 1: Used primers of lamin A/C fragments
