# Supplementary material for: Nuclear lamina strain states revealed by intermolecular force biosensor
Source: Nat Commun. 2023 Jun 30;14:3867. doi: 10.1038/s41467-023-39563-6 (PMC10313699; doi:10.1038/s41467-023-39563-6)
Supplement: Supplementary file 5 — Reporting Summary [file 41467_2023_39563_MOESM5_ESM.pdf]

nature portfolio

Corresponding author(s): Teemu Ihalainen
Daniel Conway

Last updated by author(s): Jun 2, 2023

Reporting Summary

Nature Portfolio wishes to improve the reproducibility of the work that we publish. This form provides structure for consistency and transparency in reporting. For further information on Nature Portfolio policies, see our Editorial Policies and the Editorial Policy Checklist.

Statistics

For all statistical analyses, confirm that the following items are present in the figure legend, table legend, main text, or Methods section.

n/a	Confirmed	
☐	☒	The exact sample size (*n*) for each experimental group/condition, given as a discrete number and unit of measurement
☐	☒	A statement on whether measurements were taken from distinct samples or whether the same sample was measured repeatedly
☐	☒	The statistical test(s) used AND whether they are one- or two-sided<br>*Only common tests should be described solely by name; describe more complex techniques in the Methods section.*
☒	☐	A description of all covariates tested
☐	☒	A description of any assumptions or corrections, such as tests of normality and adjustment for multiple comparisons
☐	☒	A full description of the statistical parameters including central tendency (e.g. means) or other basic estimates (e.g. regression coefficient) AND variation (e.g. standard deviation) or associated estimates of uncertainty (e.g. confidence intervals)
☐	☒	For null hypothesis testing, the test statistic (e.g. *F*, *t*, *r*) with confidence intervals, effect sizes, degrees of freedom and *P* value noted<br>*Give P values as exact values whenever suitable.*
☒	☐	For Bayesian analysis, information on the choice of priors and Markov chain Monte Carlo settings
☒	☐	For hierarchical and complex designs, identification of the appropriate level for tests and full reporting of outcomes
☐	☒	Estimates of effect sizes (e.g. Cohen's *d*, Pearson's *r*), indicating how they were calculated

Our web collection on statistics for biologists contains articles on many of the points above.

Software and code

Policy information about availability of computer code

Data collection	Zeiss LSM780 confocal was controlled with ZEN Black (v. 2012 SP5), Leica Stellaris 8 with LAS X (v. 4.4.0), Zeiss LSM710 confocal with Zen Black (v. 2011 SP7), Zeiss LSM980 with Zen Blue (v. 3.3.89.0007) and Nikon A1R+ with NIS Elements (v. 5.11.).
Data analysis	SensorFRET analysis was conducted with custom software (available at https://github.com/crmayerVCU/pySensor), riFRET was analyzed with ImageJ (v. 1.53j or higher). FLIM data was analyzed with LAS X (v. 4.4.0). Lamin-SS structure was assembled in PyMOL (version 2.5.4). Otherwise image processing was conducted with ImageJ (v. 1.53j or higher). Statistical testing and plots were done in Graphpad Prims (v. 8.x.x or higher). Figures were assembled by using Adobe Photshop (v. 22.3 or higher) and Adobe Illustrator (v. 25.1. or higher).

For manuscripts utilizing custom algorithms or software that are central to the research but not yet described in published literature, software must be made available to editors and reviewers. We strongly encourage code deposition in a community repository (e.g. GitHub). See the Nature Portfolio guidelines for submitting code & software for further information.

Data

Policy information about availability of data

All manuscripts must include a data availability statement. This statement should provide the following information, where applicable:

- Accession codes, unique identifiers, or web links for publicly available datasets
- A description of any restrictions on data availability
- For clinical datasets or third party data, please ensure that the statement adheres to our policy

The microscopy and western blot data generated in this study have been deposited in the IDA database, https://doi.org/10.23729/7f4882ab-821e-44f0-8f89-d133cc6c8dd6 under CC BY 4.0 license. The quantified data used in the figures are provided in the Supplementary Information/Source Data file. Further details of the data can be inquired from the corresponding authors.

Field-specific reporting

Please select the one below that is the best fit for your research. If you are not sure, read the appropriate sections before making your selection.

☒ Life sciences ☐ Behavioural & social sciences ☐ Ecological, evolutionary & environmental sciences

For a reference copy of the document with all sections, see nature.com/documents/nr-reporting-summary-flat.pdf

Life sciences study design

All studies must disclose on these points even when the disclosure is negative.

Sample size

The sample sizes were determined based on the previous experience of the groups regarding microscopy and the variance within the data as well as experimental constraints (e.g., how many cells can be imaged in a certain time point).

For experiments where cell populations were used as imaging data, images with multiple cells per frame were collected for at least 5 images per condition. These experiments were repeated for two to three times.
For experiments involving individual cell analysis, the experimental set up was designed to collect data from a 10-50 individual cells in at least two experimental repeats. (The exact number of cells analyzed and repeats in each experimental set-up is mentioned in the figure legends). In both cases, no statistical analysis were used to predetermine sample size.

Data exclusions

No analyzable data were excluded from analysis.

Replication

The reproducibility of the data was confirmed by repeating the experiment 2-3 times.
We also took an additional step by repeating the major experiments (FRET sensor validation) in two different laboratories with different experimental equipments and analysis methods. Although we have mentioned both the experimental set up and analysis approaches, we have only used data form either one of the laboratories in manuscript to avoid repeating the similar figures.

Randomization

Random cells or imaging frames were used in the experiments. The only exclusion criteria applied to the collected data was the overall fluorescence levels and the protein localization. This is because cells with abnormal/saturated fluorescence levels and cells with mislocalization of expressed proteins (e.g., during cell division) will lead to false results.

Blinding

Due to the typical nature of the experiments (for example adding compound during the experiment) in most of the cases, blinding was not applicable during data collection. In addition, the FRET efficiency can be detected only after the quantification step, thus user cannot influence the FRET values during acquisition.
The FRET quantification steps were conducted by using automated scripts or protocols to avoid the influence of human factor.

Reporting for specific materials, systems and methods

We require information from authors about some types of materials, experimental systems and methods used in many studies. Here, indicate whether each material, system or method listed is relevant to your study. If you are not sure if a list item applies to your research, read the appropriate section before selecting a response.

Materials & experimental systems

n/a	Involved in the study	
☐	☒	Antibodies
☐	☒	Eukaryotic cell lines
☒	☐	Palaeontology and archaeology
☒	☐	Animals and other organisms
☒	☐	Human research participants
☒	☐	Clinical data
☒	☐	Dual use research of concern

Methods

n/a	Involved in the study	
☒	☐	ChIP-seq
☒	☐	Flow cytometry
☒	☐	MRI-based neuroimaging

Antibodies

Antibodies used

Primary antibodies:
Anti-Lamin A/C antibody (Abcam (ab133256), EP4520-16), Anti-Lamin A/C antibody (Abcam (ab8984), 131C3), Anti-Lamin A/C antibody (Santa Cruz Biotechnology (sc-376248), E-1), Anti-Lamin A antibody (Santa Cruz Biotechnology (sc-7292), 636), Anti-Histone H3K27ac antibody (Abcam (ab4729)), Anti-Histone 2A antibody (Cell Signaling (D6O3A), 12349S). Anti-Actin antibody (clone C4, Merk-Millipore (MAB1501R)).
Secondary antibodies:
Goat anti-mouse DyLight 800 (Thermo Scientific (SA5-10176)), Goat anti-rabbit DyLight 680 (Thermo Scientific (35568)), Goat anti-mouse Alexa 488 (Thermo Scientific (A-11001)), Goat anti-rabbit Alexa 568 (Thermo Scientific (A-11004)), Goat anti-mouse Alexa 647

	(Thermo Scientific (A-21235))
Validation	Lamin A/C antibody EP4520-16 was knock-out validated by the manufacturer. Lamin A/C 636, Histone 2A D603A and Histone H3K27ac antibodies were validated by the manufacturer in ChIP. Lamin A/C antibodies E-1 and 131C3 were tested in lamin KO cells by Ihalainen lab.

Eukaryotic cell lines

Policy information about cell lines

Cell line source(s)	MDCK II (obtained from Jennifer Lippincott-Schwartz lab) was used for all experiments, except MDCK II (obtained from Aki Manninen lab (University of Oulu, Finland), originally from ECACC #00062107) was used for lamin A knockout experiments.
Authentication	The cell lines were not authenticated by the researchers.
Mycoplasma contamination	Cells were routinely tested for mycoplasma.
Commonly misidentified lines (See ICLAC register)	None used
